# Supplementary material for: Elexacaftor Mediates the Rescue of F508del CFTR Functional Expression Interacting with MSD2
Source: Int J Mol Sci. 2023 Aug 16;24(16):12838. doi: 10.3390/ijms241612838 (PMC10454486; doi:10.3390/ijms241612838)
Supplement: Supplementary file 1 [file ijms-24-12838-s001.zip › ijms-2513438-supplementary.pdf]

Elexacaftor Mediates the Rescue of F508del CFTR Functional Expression Interacting with the MSD2

Roberta Bongiorno [1], Alessandra Ludovico [1], Oscar Moran [1] and Debora Baroni [1,*]

[1] Istituto di Biofisica, CNR. Via De Marini, 6. 16149 Genova, Italy
* **Correspondence: debora.baroni@ibf.cnr.it; Tel. +39-0106475559. Fax +39-0106475500**

Supplementary materials

Table S1. Evaluation of CFTR mRNA abundance in HEK-t cells transfected with WT and F508del CFTR after treatment with 5 µM VX809, 5 µM VX661, 10 µM CORR4A and 5 µM VX445 correctors. The mRNA abundance is expressed as the cycle threshold (Ct) obtained by real-time PCR and normalized to the expression of the untreated samples (control condition) and the of GAPDH, used as housekeeping gene. Data represent the mean ± sem (standard error of the mean). For each condition, the number of observations is indicated into the brackets. Comparisons of values with those of control (untreated) cells were made with the Dunnett's test. Probability p values are reported. Differences are significant when $p < 0.05$.

	**WT CFTR**		**F508del CFTR**	
	**normalised Ct**	**p**	**normalised Ct**	**p**
**Untreated**	1.00 ± 0.20 (18)		1.00 ± 0.18 (10)	
**+ VX809**	1.11 ± 0.14 (9)	0.14	1.00 ± 0.01 (13)	0.10
**+ VX661**	1,10 ± 0.14 (9)	0.28	0.98 ± 0.03 (13)	0.17
**+ CORR4A**	1,08 ± 0.14 (9)	0.21	0.97 ± 0.13 (12)	0.19
**+VX445**	1.12 ± 0.16 (18)	0.26	1.05 ± 0.17 (9)	0.44

Table S2. Evaluation of MSD1, WT and F598del NBD1, R-domain, MSD2 and NBD2 mRNA abundance in HEK-t cells transfected with MSD1, WT- and F598del NBD1, R-domain, MSD2, or NBD2 mRNAs after treatment with 5 µM VX809, 5 µM VX661, 10 µM CORR4A and 5 µM VX445. The mRNA abundance is expressed as the cycle threshold (Ct) obtained by real-time PCR and normalized to the expression of the untreated samples (control condition) and of GAPDH, used as housekeeping gene. Data represent the mean ± sem (standard error of the mean). For each condition, the number of observations is indicated into the brackets. Comparisons of values with those of control (untreated) cells were made with the Dunnett's test. Probability p values are reported. Differences are significant when $p < 0.05$.

	**MSD1**		**WT-NBD1**		**F508del-NBD1**	
	**normalised Ct**	**p**	**normalised Ct**	**p**	**normalised Ct**	**p**
**Untreated**	1.00 ± 0.21 (10)		1.00 ± 0.15 (13)		1.00 ± 0.33 (12)	
**+ VX809**	1.03 ± 0.17 (9)	0.83	1.00 ±0.20 (9)	0.55	1.02 ± 0.27 (13)	0.52
**+ VX661**	1.06 ± 0.11 (8)	0.79	1.01 ±0.18 (17)	0.08	0.97 ± 0.28 (13)	0.52
**+ CORR4A**	1.01 ± 0.18 (9)	0.82	1.00 ±0.16 (8)	0.87	0.97 ± 0.17 (12)	0.36
**+VX445**	1.08 ± 0.22 (9)	0.40	1.07 ±0.11 (13)	0.06	1.06 ± 0.20 (14)	0.31
	**R domain**		**MSD2**		**NBD2**	
	**normalised Ct**	**p**	**normalised Ct**		**normalised Ct**	**p**
**Untreated**	1.00 ± 0.18 (9)		1.00 ± 0.27 (16)	0.59	1.00 ± 0.13 (7)	
**+ VX809**	0.95 ± 0.13 (9)	0.13	1.05 ± 0.22 (18)	0.07	0.99 ± 0.20 (10)	0.24
**+ VX661**	1.01 ± 0.22 (8)	0.37	1.08 ± 0.20 (20)	0.16	1.09 ± 0.18 (11)	0.06
**+ CORR4A**	1.10 ± 0.22 (8)	0.07	1.08 ± 0.22 (18)	0.16	1.00 ± 0.11 (10)	>0.99
**+VX445**	1.02 ± 0.13(13)	0.26	1.03 ± 0.21 (20)	0.65	1.06 ± 0.09 (12)	0.30

Table S3. Evaluation of F508del CFTR protein expression in HEK-t cells whole-cell lysates after treatment with 5 µM VX809, 5 µM VX661, 10 µM CORR4A and 5 µM VX445. Quantification either of total protein (C + B bands) and maturation rate (C/(C+B) bands) was obtained analyzing the band intensity with the software imageJ. Retrieved values were normalized to the intensity of the protein actin used as housekeeper protein and to the expression value of untreated F508del protein. Data represent the mean ± sem (standard error of the mean). For each condition, samples were run at least in quadruplicate. Comparisons of values with those of control, untreated samples were made with the Dunnett's test. Probability p values are reported. Asterisks indicate a significant difference ($p < 0.05$).

**WT CFTR**					
	**Control**	**VX809**	**VX661**	**CORR4A**	**VX445**
**C+B**	1.00 ± 0.11	1.01 ± 0.19<br>P = 0.16	1.17 ± 0.17<br>P = 0.25	1.12 ± 0.19<br>P = 0.28	1.04 ± 0.11<br>P = 0.11
**C/(C +B)**	1.00 ± 0.01	1.03 ± 0.09<br>P = 0.14	1.18 ± 0.17<br>P = 0.29	1.16 ± 0.14<br>P = 0.38	1.01 ± 0.23<br>P = 0.17
**F508del CFTR**					
	**Control**	**VX809**	**VX661**	**CORR4A**	**VX445**
**C+B**	1.00 ± 0.07	2.84 ± 0.16<br>P = 0.016 (*)	2.75 ± 0.15<br>P = 0.015 (*)	2.48 ± 0.22<br>P = 0.018 (*)	3.00 ± 0.18<br>P = 0.018 (*)
**C/(C +B)**	1.00 ± 0.01	2.09 ± 0.09<br>P = 0.006 (*)	2.05 ± 0.10<br>P = 0.005 (*)	1.92 ± 0.11<br>P = 0.024 (*)	2.35 ± 0.12<br>P = 0.07 (*)

Table S4. Evaluation of CFTR single domain expression in HEK-t cells whole-cell lysates after treatment with 5 µM VX809, 5 µM VX661, 10 µM CORR4A and 5 µM VX445. Quantification protein expression was obtained analyzing the band intensity with the software imageJ. Retrieved values were normalized to the intensity of the protein actin used as housekeeper protein and to the expression value of each untreated single domain. Data represent the mean ± sem (standard error of the mean). For each condition, samples were run at least in quadruplicate. Comparisons of values with those of control, untreated samples were made with the Dunnett's test. Probability p values are reported. Asterisks indicate a significant difference ($p < 0.05$).

CFTR domain	Control	VX809	VX661	CORR4A	VX445
**MSD1**	1.00 ± 0.08	2.69 ± 0.12<br>P = 0.004 (*)	2.61 ± 0.14<br>P = 0.005 (*)	1.04 ± 0.10<br>P = 0.33	1.08 ± 0.12<br>P = 0.19
**WT NBD1**	1.00 ± 0.12	0.98 ± 0.13<br>P = 0.59	1.03 ± 0.12<br>P = 0.28	0.97 ± 0.09<br>P = 0.40	0.97 ± 0.09<br>P = 0.33
**F508del NBD1**	1.00 ± 0.04	1.03 ± 0.08<br>P = 0.19	0.96 ± 0.10<br>P = 0.29	1.05 ± 0.07<br>P = 0.09	1.07 ± 0.09<br>P = 0.59
**R domain**	0.99 ± 0.11	1.11 ± 0.12<br>P = 0.28	1.04 ± 0.10<br>P = 0.48	1.02 ± 0.12<br>P = 0.58	1.12 ± 0.08<br>P = 0.09
**NBD2**	1.01 ± 0.07	0.99 ± 0.13<br>P = 0.63	1.01 ± 0.11<br>P = 0.54	2.34 ± 0.13<br>P = 0.02 (*)	1.02 ± 0.11<br>P = 0.40
**MSD2**	1.01 ± 0.10	1.01 ± 0.10<br>P = 0.25	0.99 ± 0.09<br>P = 0.41	0.97 ± 0.17<br>P = 0.74	2.88 ± 0.13<br>P = 0.018 (*)

Table S5. Evaluation of MSD2 protein expression in HEK-t cells whole-cell lysates after treatment with correctors and protein new synthesis blockage with cycloheximide. Quantification of protein expression was obtained analyzing the band intensity with the software imageJ. Retrieved values were normalized to the intensity of the protein actin used as housekeeper protein and to the value of MSD2 protein expression at time 0. Data represent the mean ± sem (standard error of the mean). For each condition, samples were run at least in quadruplicate. Comparisons of values with those of control, untreated samples were made with the Dunnett's test. Probability p values are reported. Asterisks indicate a significant difference ($p < 0.05$).

time (hours)	Control	VX809	VX661	CORR4A	VX445
**0**	1.00 ± 0.01	1.00 ± 0.02<br>P = 0.41	1.00 ± 0.03<br>P = 0.23	1.00 ± 0.03<br>P = 0.68	1.00 ± 0.03<br>P = 0.33
**1**	0.88 ± 0.06	0.90 ± 0.04<br>P = 0.25	0.92 ± 0.03<br>P = 0.17	0.89 ± 0.01<br>P = 0.52	0.93 ± 0.04<br>P = 0.26
**2**	0.72 ± 0.02	0.73 ± 0.02<br>P = 0.32	0.76 ± 0.07<br>P = 0.23	0.72 ± 0.04<br>P = 0.53	0.84 ± 0.04<br>P = 0.65
**4**	0.41± 0.01	0.42 ± 0.04<br>P = 0.11	0.47 ± 0.03<br>P = 0.24	0.44 ± 0.07<br>P = 0.11	0.59 ± 0.02<br>P <b0.01 (*)
**6**	0.30 ± 0.02	0.31 ± 0.06<br>P = 0.17	0.33 ± 0.04<br>P = 0.24	0.32± 0.02<br>P = 0.68	0.44 ± 0.01<br>P < 0.02 (*)
**8**	0.25 ± 0.03	0.26 ± 0.04<br>P = 0.31	0.28 ± 0.03<br>P = 0.53	0.25 ± 0.05<br>P = 0.41	0.31 ± 0.02<br>P = 0.82

Table S6. Primary antibodies used to detect whole length F508del CFTR and CFTR single domains used in this work. For each antibody it is indicated the vendor, the clonality, their diluition (in PBS tween20 + 5% albumin) used to perform the western blot experiments, the epitope (when available in the data sheet) and the CFTR domain detected in our experiments

Antibody identifier	Vendors	Clonality	Diluition	Epitope	Target identified:
MM13-4	Millipore	Mouse monoclonal	1:200	N-terminus; residues 25-36	Full-lenght CFTR and MSD1
L12B4	Millipore	Mouse monoclonal	1:200	Residues 386-412	WT/F508del NBD1
Clone # 13-1 MAB1660	R&D system	Mouse monoclonal	1:100	R domain	R domain
ABIN350208	Antibodies on line	Rabbit polyclonal	1:100	Residues1150–1200	MSD2
clone M3A7	Millipore	Mouse monoclonal	1:200	C-terminus; residues 1365-1395	NBD2
